# Calculation of the Point of Application (Centre of Pressure) of Force and Torque Imparted on a Spherical Object from Gyroscope Sensor Data, Using Sports Balls as Practical Examples

**DOI:** 10.3390/s24175810

**Published:** 2024-09-07

**Authors:** Franz Konstantin Fuss, Batdelger Doljin, René E. D. Ferdinands

**Affiliations:** 1 Chair of Biomechanics, Faculty of Engineering Science, University of Bayreuth, D-95447 Bayreuth, Germany; franzkonstantin.fuss@uni-bayreuth.de; 2 Division of Biomechatronics, Fraunhofer Institute for Manufacturing Engineering and Automation IPA, D-95447 Bayreuth, Germany; 3 Smart Products Engineering Program, Swinburne University, Melbourne, VIC 3000, Australia; batdelger.doljin@gmail.com; 4 Discipline of Exercise and Sports Science, University of Sydney, Sydney, NSW 2141, Australia; edouard.ferdinands@sydney.edu.au

**Keywords:** smart ball, IMU gyroscope, centre of pressure, cricket, football, spin rate, torque, signal noise, cross-product, noise management

## Abstract

This study investigates the determination of the centre of pressure (COP) on spherical sports objects such as cricket balls and footballs using gyroscope data from Inertial Measurement Units (IMUs). Conventional pressure sensors are not suitable for capturing the tangential forces responsible for torque generation. This research presents a novel method to calculate the COP solely from gyroscope data and avoids the complexity of isolating user-induced accelerations from IMU data. The COP is determined from the cross-product of consecutive torque vectors intersecting the surface of the sphere. Effective noise management techniques, including filtering and data interpolation, were employed to improve COP visualisation. Experiments were conducted using a smart cricket ball and a smart football. Validation tests using spin rates between 7.5 and 12 rps and torques ranging from 0.08 to 0.12 Nm confirmed consistent COP clustering around the expected positions. Further analysis extended to various spin bowling deliveries recorded using a smart cricket ball, and a curved football kick recorded using a smart football demonstrated the wide applicability of the method. The COPs of various spin bowling deliveries showed adjacent positions on the surface of the ball, traversing through backspin, sidespin and topspin, excluding the flipper and doosra deliveries. The calculation of the COP on the surface of the soccer ball could only be achieved by increasing the data sampling frequency sevenfold using curve fitting. Knowledge and use of the COP position offers significant advances in understanding and analysing ball dynamics in sports.

## 1. Introduction

Understanding the determination of the centre of pressure (COP) on spherical objects is critical for analysing the dynamics involved in ball-related sports. The COP is an essential characteristic that provides insight into the behaviour of a ball during and after interaction with a player either through a part of the body or an implement acting as the end-effector. This includes insights on the various types of forces and torques that are applied, influencing the trajectory, spin and stability of the ball. Despite the complexity of these interactions, accurately identifying the COP has significant implications for both performance optimisation and training in sports such as cricket and football.

Traditional methods for determining the centre of pressure (COP) on spherical objects often rely on pressure sensors distributed on the surface of the ball that measure the location of the COP directly. For example, Yeh et al. [1] used two pressure sensors mounted on a baseball. However, the placement of two pressure sensors is not sufficient to calculate the COP, specifically when two fingers are placed on these sensors. While useful, these methods only calculate the normal force from the pressure but do not capture the full range of forces involved, particularly the tangential forces (or friction forces) that generate torque. Torque plays a crucial role in the ball’s behaviour, especially in spin and flight dynamics. Hence, a more comprehensive method that includes these tangential forces is required for a precise determination of the COP.

Another method uses the acceleration signal of an Inertial Measurement Unit (IMU) to calculate the direction of the normal force imparted on a ball, after isolating the user-induced acceleration (UIA) component and converting it to force [2]. However, isolating the UIA from the measured acceleration of an IMU is difficult, as the measured acceleration includes other accelerations as well, such as the gravitational, centripetal and Coriolis acceleration vectors, pointing in different directions.

The importance of understanding the COP is clearly illustrated by a simple example from seam bowling in cricket. According to Woolmer and Noakes [3], “*the first two fingers rest on either side of the seam*” and the “*first and second fingers impart equal amounts of back-spin to the ball*”. This statement would imply that the point of application (called the centre of pressure, COP) of force and torque imparted on the cricket ball must be exactly on the seam. However, Fuss et al. [4,5], using a smart cricket ball, showed that this statement is mechanically incorrect, as the COP “*should be approximately 10–15° off the seam in order to avoid seam wobble*”. The basis of this principle is that the angular velocity vector **ω** moves towards the resultant torque vector **T_R_** [5,6] imparted on the ball, but **ω** cannot reach **T_R_** before the ball is released. To ensure that the **ω**-vector is precisely at the pole of the ball (to prevent seam wobbling), the **T_R_**-vector and the corresponding COP must be outside the pole and seam, respectively. Knowing the exact position of the COP is therefore of utmost importance for training purposes when using a smart ball.

Cricket and football were chosen for this study due to the strategic application of the Magnus force in both sports. In cricket, this is evident in the swerve of the ball, whereas, in football, it is seen in the curved kick. The influence of the Magnus force increases in proportion to the spin rate (*ω*).

This raises the question of whether the COP can be calculated only from the angular velocity signal ω without relying on the translational acceleration signal. This study aims to address this question by exploring solutions for calculating and visualising the position of the COP on a spherical object from gyroscope sensor data, obtained from a smart cricket ball during various types of spin deliveries and from a smart football during kicking.

## 2. Materials and Methods

### 2.1. Mechanical Principles

This study focuses on processing only the gyroscope data from Inertial Measurement Units (IMUs) embedded in smart sports balls to calculate the COP. For instance, the smart cricket ball and smart football are equipped with high-speed rate gyros capable of capturing the angular velocity of the ball at high resolutions [4,6]. The objective of this approach is to explore a novel methodology that overcomes the limitations of traditional pressure sensors and provides a more accurate and detailed analysis of the COP. Since the primary challenge in determining the COP from gyroscope data lies in accurately interpreting the tangential forces and the resultant torques, we explored the relationship between the tangential force, the torque it produces and the geometry of the sphere using an abstract geometrical approach, where the torque vector and the radius of the sphere are conceptualised as a plane that intersects the surface at a great circle. The COP, which is the origin of both the normal and tangential forces, is therefore located somewhere along this great circle. However, the direction of the torque vector is not stationary, especially in dynamic conditions like those produced by human movements, so the COP must move continuously. This continuous movement enables the determination of the COP by examining the intersection of great circles formed by consecutive torque vectors.

**Figure 1:**
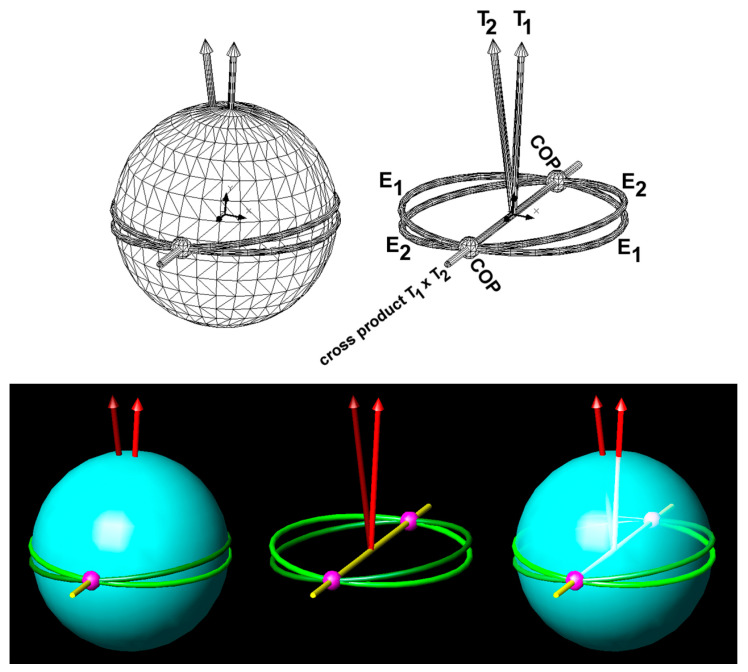
Sphere with two consecutive torque vectors. COP (magenta): centre of pressure; T_1_ (light red) and T_2_ (dark red): two torque vectors; E_1_ (light green) and E_2_ (dark green): great circles associated with T_1_ and T_2_; yellow line: cross-product vector of T_1_ and T_2_, connecting the two COPs.

When force and torque are imparted to a sphere, the normal component **F_N_** of the resultant force vector is perpendicular to the surface and passes through the centre of mass (COM). The tangential component of the resultant force vector is the friction force **F_F_**, which produces the torque **T**:

(1) $$T=F_{F}\times R$$

where **R** is the radius of the sphere. The vectors **F_F_** and **R** form a plane that passes through the COM, which is perpendicular to **T**, and that intersects the surface of the sphere in a great circle. The COP, the origin of **F_N_** and **F_F_**, therefore lies along a great circle. Even if the direction of **T** is known, the position of the centre of pressure (COP) cannot be determined, as it could be located anywhere along the associated great circle. Thus, there are infinitely many possible solutions for the COP. However, the position of the **T**-vector is not stationary, at least not when generated by human movements. Considering that the **T**-vector moves continuously, and the COP moves even without sudden jumps, e.g., when a ball rolls along or over the curved fingers or along the medial edge of a football shoe, then **two consecutive T-vectors must share the same COP**. This principle is illustrated in Figure 1. Specifically, if the great circles E_1_ and E_2_ (Figure 1) are associated with vectors **T**_1_ and **T**_2_, respectively, then the COP is located at the intersections of E_1_ and E_2_. Naturally, there are two intersections, which means that the previously assumed infinite number of COP solutions is reduced to two solutions, namely two points antipodal to each other, which are therefore conjugate points. Essentially, the intersections of the two great circles (i.e., the two COPs) are calculated from the cross-product of the two torque vectors (Figure 1):

(2) $$C=T_{1}\times T_{2}$$

where **C** is the vector from the centre of the sphere to the COP.

Note that the COP cannot be determined from the **ω**-vectors. It is tempting to use the same method as above—namely, to calculate the cross-product of two consecutive **ω**-vectors (since the tangential force is supposed to change the amount of spin rate anyway). However, the direction of the **ω**-vector is not directly dependent on the tangential force, unlike the direction of the **T**-vector. The reason for this is that the tangential force produces a torque vector in a specific direction. This resultant **T**-vector has two components with respect to the **ω**-vector present at the same time. These two components are a component parallel to the **ω**-vector (the spin torque **T_S_**) and a component perpendicular to the **ω**-vector (the precession torque **T_P_**) [6]. **T_S_** produces the angular acceleration **α**, which changes the magnitude of the angular velocity **ω**:

(3) $$\alpha =\frac{T_{S}}{I}$$

where *I* denotes the moment of inertia.

**T_P_** causes a movement (tilt or rotation) of the spin axis **ω**, commonly known as precession *p*, with the unit of rad/s (angular velocity of the movement of the spin axis **ω**, not to be confused with the angular velocity of the object):

(4) $$p=\frac{T_{P}}{\omega \;I}$$

The sense of the spin axis rotation is in the direction of the **T**-vector, whereby the **ω**-vector rotates about the precession vector **p**. The movement of the spin axis (**ω**-vector) is therefore caused by the direction of the **T**-vector and not by the tangential force **F_F_**. The cross-product of two **ω**-vectors therefore has no relation to the COP but provides the exact direction of the precession vector **p**.

### 2.2. Sensor Data and Signal Processing

**Figure 2:**
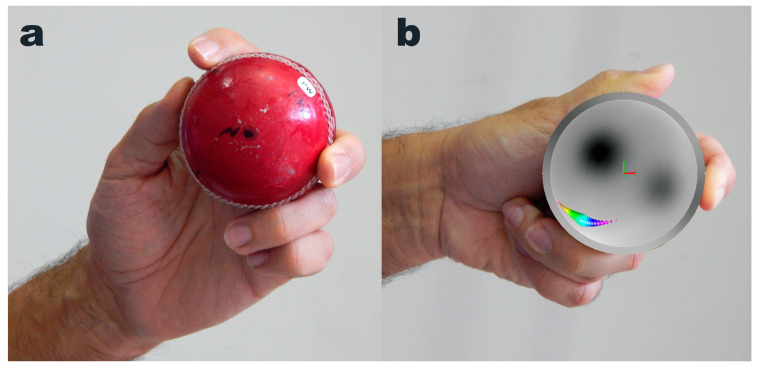
Experimental procedure: position of the hand and smart cricket ball at (**a**) the beginning of angularly accelerating the ball and (**b**) just before the release. (**b**) The ball is replaced by a hollow hemisphere, showing the position of the COP (in rainbow colours) at the contact between the middle finger and the ball.

The smart cricket ball (Figure 2) used in this study was developed by Fuss et al. in late 2011 [4]. This ball is instrumented with three high-speed rate gyros that measure angular velocity up to 20,000°/s (55 rps) [4,6]. To identify the correct position of the COP with respect to the hand and the ball/sensor coordinate system, the index finger was placed on the seam at +X (xy-plane corresponds to the plane of the seam) and +Z (north pole of the ball) pointed out of the hand in left-handed players and into the palm in right-handed players. To determine the position of the COP with respect to the ball during 15 different spin bowling deliveries, a left-handed former first-class spin bowler (REDF) bowled the ball six times per delivery (Figure 2). The ball records the angular velocity data at 815 Hz and transmits them wirelessly to a laptop or smartphone. After downloading the data, we processed them with our smart cricket ball software [6]. The angular velocity data were filtered with a third-order low-pass Butterworth filter at a cutoff frequency of 33 Hz. The resultant angular velocity *ω* was calculated from the individual *ω_x_*, *ω_y_* and *ω_z_* components obtained from each sensor:

(5) $$\omega_{R}=\sqrt{{\omega_{x}}^{2}+{\omega_{y}}^{2}+{\omega_{z}}^{2}}$$

The components and the resultant angular acceleration resulted from

(6) $$\alpha_{xyz}=\frac{d\omega_{xyz}}{dt}$$

and

(7) $$\alpha_{R}=\sqrt{{\alpha_{x}}^{2}+{\alpha_{y}}^{2}+{\alpha_{z}}^{2}}$$

The components and the resultant torque were calculated from

(8) $$T_{xyz}=I_{xyz}\alpha_{xyz}+\left(I_{zxy}-I_{yzx}\right)\;\omega_{yzx}\;\omega_{zxy}$$

where *I* denotes the moment of inertia of the ball, and *I_x_* ≈ *I_y_* ≈ *I_z_*, and

(9) $$T_{R}=\sqrt{{T_{x}}^{2}+{T_{y}}^{2}+{T_{z}}^{2}}$$

The position of the COP was calculated from Equation (2). Of the two COP solutions, the correct COP position is expected in the negative Y hemisphere for left-handed individuals and in the negative Z hemisphere (closer to the palm for left-handed individuals).

The football data were obtained from a curved kick of the IOTIS smart football (IOTIS GmbH, Hannover, Germany). When executing the curve kick, the player struck the ball off-centre, using the inside of his foot, applying both a forward force and a tangential force along the side of the ball, causing it to spin and inducing the Magnus force, which causes a lateral deviation in ball flight. The IOTIS ball is instrumented with an IMU that measures the angular velocity up to 4000°/s with a triaxial gyro, translational acceleration up to 16 g with a triaxial accelerometer and magnetic field strength with a triaxial magnetometer. The IMU data were recorded with 1024 Hz. The angular velocity data were used to investigate whether the COP can be calculated during the short contact time of 7–8 ms between shoe and ball. The angular velocity data were processed in the same way as those of the smart cricket ball. Orienting the ball to the shoe or to the global coordinate system was impossible, as the exact orientation of the sensor coordinate system was unknown. The sensors were sandwiched in the IOTIS smart football between the bladder and the outside.

### 2.3. Methods for Noise Management

**Figure 3:**
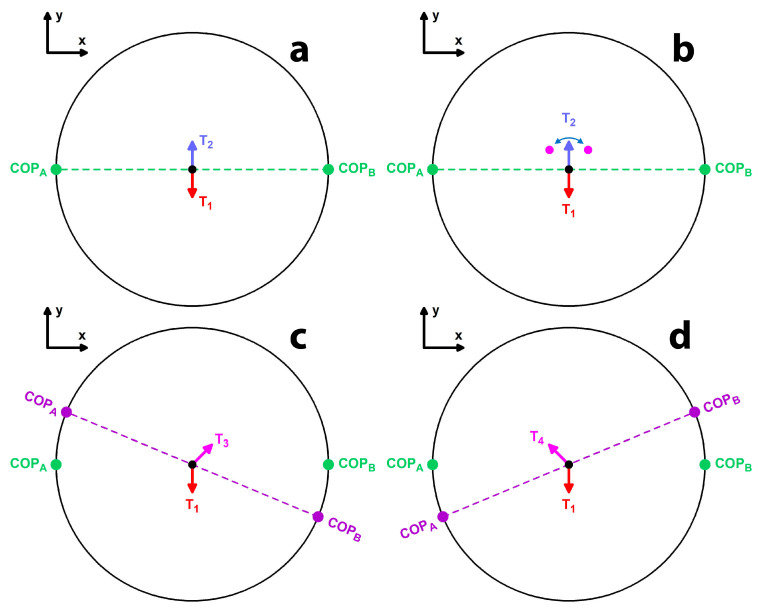
Explanation of the circumferential noise (top view of a sphere): (**a**) two consecutive unit vectors (T_1_ and T_2_) of a torque provide two solutions of the COP (COP_A_ and COP_B_); the dashed green line is the cross-product of T_1_ and T_2_. (**b**) T_2_ is affected by noise and thereby deflected sideways in the ±x-direction where the two magenta dots are located. (**c**) If T_2_ becomes T_3_, then the cross-product vector rotates clockwise (purple dashed line) and the COP jumps along the great circle (black). (**d**) If T_2_ becomes T_4_, then the cross-product vector rotates counterclockwise and the COP jumps along the great circle in the opposite direction.

The intersection of two great circles is affected by noise in the form of “circumferential noise”. This means that, although the COP cannot move perpendicular to the great circles, it can certainly move along them, jumping forward and backward on the great circle. This noise is generated by the torque vector moving along its path on the sphere’s surface as it suddenly deviates sideways and oscillates around its path (Figure 3). The smaller the torque magnitude is, the larger these circumferential errors are.

There are five methods to solve this problem:

(1) filtering of the raw *ω* data, such as average filters (including higher-order Savitzky–Golay filters), low-pass filters, polynomial fits, etc.;
(2) reduction of the COP movement by raw data interpolation (simulation of a higher data sampling frequency);
(3) calculating the cross-product not from consecutive T-vectors but from a set of two vectors separated by more than one vector;
(4) confining the COP data to large torques;
(5) finally filtering the path of the COP on the surface of the sphere or ball.

These five methods were implemented in our smart cricket ball software as follows. Filtering of the angular velocity data of the smart cricket ball with a third-order Butterworth low-pass filter at a cutoff frequency of 33 Hz was mentioned above. The angular velocity data of the smart football were fitted with sixth-order polynomials over the angular acceleration phase. The data sampling frequency was virtually increased by a factor of two, i.e., to 1630 Hz for the smart cricket ball. For the smart football, the frequency was set to 7500 Hz, and the angular velocity data *ω_xyz_* were calculated from the new timestamp and the polynomial fit functions. The cross-product was calculated from a set of two vectors separated by 10 data. The last two methods are related to visualising the COP and its path, e.g., in 2D on a graph, or in 3D on a sphere, where the individual COPs are represented as bubbles (circle in 2D and sphere in 3D) sized to the magnitude of the normalised torque T_n_ = T/T_max_ or powers thereof, e.g., T_n_^C^, where the exponent c is an integer. The peak of T_n_ still remains 1, but T_n_ data smaller than 1 are suppressed more or less, depending on their size. Higher powers, e.g., c ≥ 4, provide acceptable results. The final path of the COP on the surface of the ball was smoothed with a second-order Savitzky–Golay filter with a window width of 10 data.

**Figure 4:**
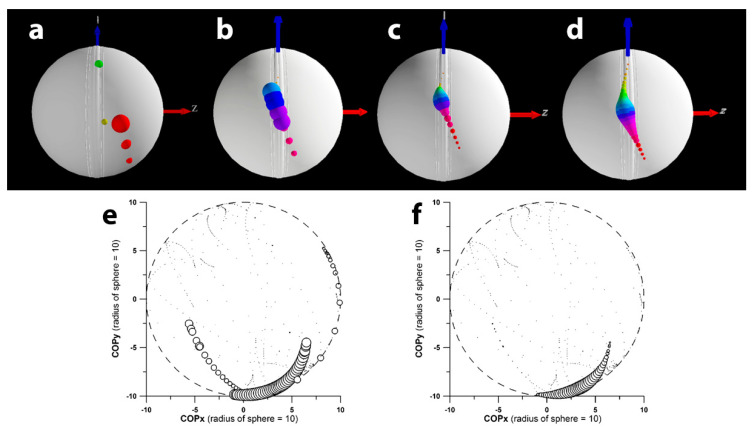
Effect of noise management exemplified in the smart cricket ball: (**a**) COP (centre of pressure) calculated from the raw data; (**b**) COP after filtering; (**c**) COP after data interpolation and calculation of the COP from torque data that were 10 data points apart; (**d**) bubble size of the COP corresponding to the magnitude of the normalised torque T_n_ to the power of eight (T_n_**^8^**); in subfigures (**a**–**d**) the COP-bubbles are colour-coded to indicate the direction of the COP-movement (red-yellow-green-blue-magenta); (**e**,**f**) exemplify the effect of controlling the bubble size of the COP: (**e**) T_n_**^1^** and (**f**) T_n_**^8^**.

It is beneficial to colour-code the COP bubbles (Figure 2 and Figure 4), with the hue corresponding to the timestamp of the raw angular velocity data. For example, the time period from the start time to the end time is encoded with sequential colours from red through orange, yellow, green, blue, and finally purple. The colour coding helps to visualise the direction of the COP path. For example, if a ball rolls out of a person’s hand in a counterclockwise direction from an observer’s perspective, the COP will move clockwise relative to, and on the surface of, the ball from that same perspective.

### 2.4. Validation Study

The seam of the smart cricket ball was placed exactly on the positive x-axis of the ball coordinate system on the tip of the index finger. A small drop of glue was applied to the seam at +x and allowed to dry so that the position of +x could be felt on the tip of the index finger. The ball was initially stabilised by the index finger and thumb of the opposite hand. The ball was then rapidly accelerated upwards by imparting topspin to the ball with the index finger (torque vector on the +z-axis) while simultaneously rolling the ball over the tip of the index finger. This experiment was repeated 100 times. The COP position at the torque peak was used to determine the deviation of the COP from the +x-axis.

## 3. Results

### 3.1. Noise Management

Figure 4 shows the improvement in COP calculation and visualisation when the noise component is effectively managed. The data shown result from the validation tests where the COP is expected at +x (1,0,0). Figure 4abcd shows the evolution of improvement when implementing the different methods one by one (filtering the path of the COP on the surface of the sphere or ball was not required, because the path was smooth to begin with). Figure 4ef shows how deviating COP locations are suppressed by modulating the bubble size of the COP with the exponent of the normalised torque (T_n_^C^).

### 3.2. Validation

**Figure 5:**
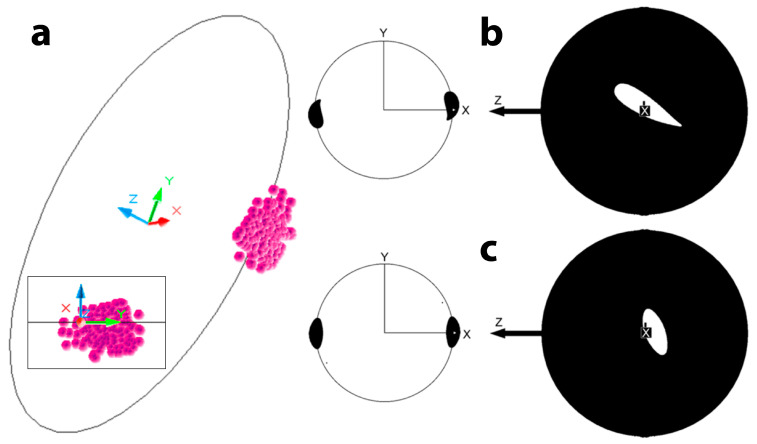
Results of the validation test: (**a**) 3D iso view of 100 COP positions (inset: projection on the yz-plane); (**b**,**c**) examples of COP footprints in the xy- and yz-planes.

The maximum spin rate of the validation experiments ranged from 7.5 to 12 rps, and the maximum torque ranged from 0.08 to 0.12 Nm. Figure 5 shows the results of the validation test. Figure 5a identifies the 3D COP positions at the peak torque, expected at R,0,0 (R is the radius of the smart cricket ball; 36 mm). The averages (±standard deviation) of the COP cluster in the y- and z-directions were +3.0 ± 3.6 mm and −1.2 ± 2.5 mm, respectively (Figure 5a, inset). Figure 5b,c show the footprints of two moving COPs projected on the xy- and yz-planes.

### 3.3. COP Positions of Spin Bowling Deliveries in Cricket

The average spin rates *ω* and torques *T* of the deliveries used for calculating the COP are shown in Table 1.

**Figure 6:**
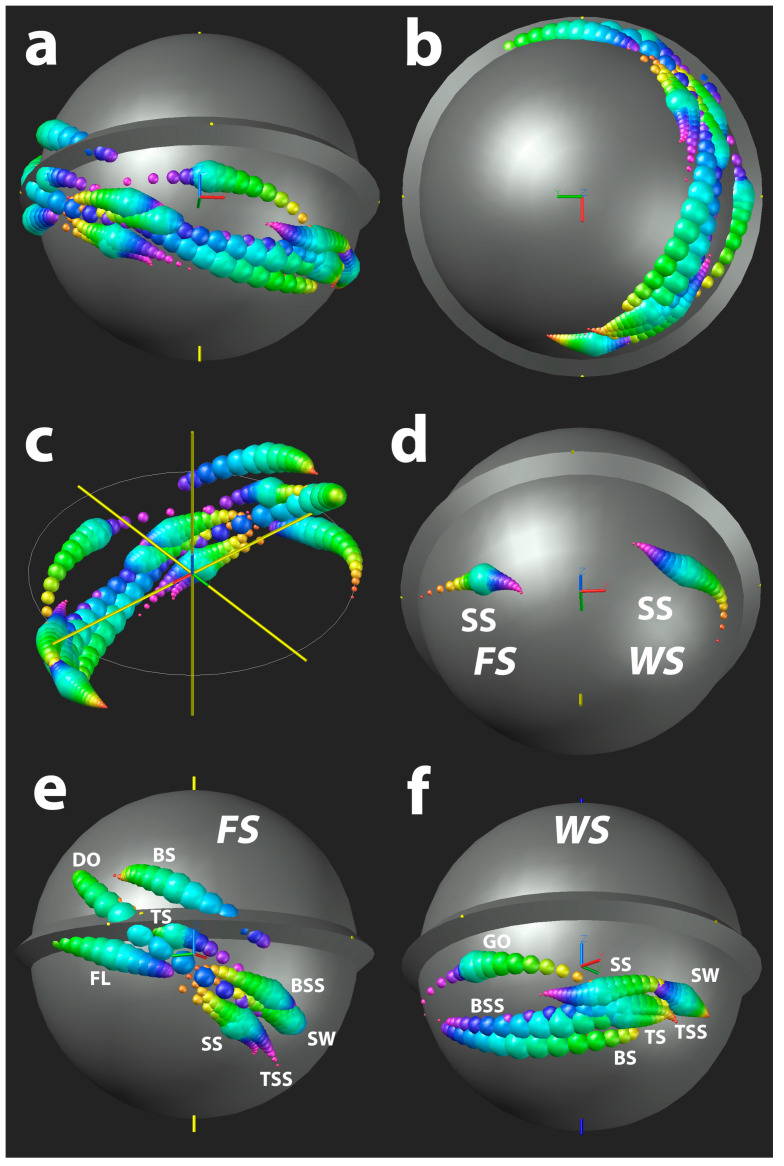
Average positions of the different spin bowling deliveries: (**a**) all 13 deliveries (Table 1) in a slightly tilted front view (the lower hemisphere is inside the palm of a left-handed spin bowler); (**b**) all 13 deliveries in a palmar view; (**c**) opposite view of (**a**) after removal of the ball (which would block the view); (**d**) COP positions of the sidespin (SS) deliveries for finger spin (FS) and wrist spin (WS); (**e**) family of finger spin deliveries (FL: flipper, BS: back spin, BSS: back side spin, SW: swerve, TSS: top side spin, TS: top spin and DO: doosra); (**f**) family of wrist spin deliveries (GO: googly).

Figure 6 shows the COP locations of the different deliveries identified in Table 1.

### 3.4. COP Calculation When Kicking a Smart Football

**Figure 7:**
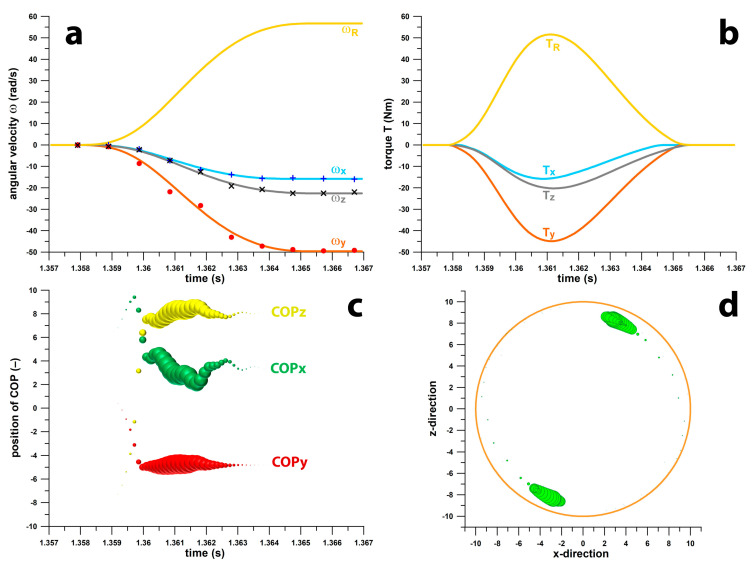
Smart football data: (**a**) angular velocity ω (rad/s) vs. time (s); xyz-components (ω_x_, ω_y_ and ω_z_) and resultant ω_R_; raw data (+, ×, •) and 6th-order polynomial fit functions (solid lines); (**b**) torque T (Nm) vs. time (s), xyz-components (T_x_, T_y_ and T_z_) and resultant T_R_; (**c**) unit vector data of the COP vs. time (s) calculated from T_n_**^8^** (bubble size = T_n_**^8^**); (**d**) COP locations (green) projected on the xz-plane of the ball (orange).

Figure 7 shows the *ω*, *T*, and COP data for the curved kick. The maximum spin rate (Figure 7a) was 56.73 rad/s (9.03 rps), and the maximum torque was 51.61 Nm (Figure 7b). The peak torque seems to be unreasonably high, but its magnitude is logical when considering that the ball had to accelerate from 0 to 56.73 rad/s within a time window of merely 7.6 ms. The average resultant torque over this window was 25.9 Nm, corresponding to an average angular acceleration of 7463.9 rad/s^2^. The latter multiplied by 0.0076 s gives 56.73 rad/s. Figure 7c shows the unit vectors vs. time, with the bubble size equal to T_n_**^8^**, which is approximately the top quarter of the resultant torque. Figure 7d shows the two solutions (conjugate points) of the COP projected on the xz-plane. The COP moves approximately 20° on the surface of the ball.

## 4. Discussion

To the best of the authors’ knowledge, this is the first time that the cross-product of two torque vectors has been used to calculate the point on a spinning spherical object where the torque was applied. In addition, this study is a typical example of indirect sensing, because the COP was not measured using a pressure sensor array. There are basically two ways for indirect sensing:

- an object—or more precisely, the material of an object—is the sensor itself, instead of embedding a sensor into the object;
- the data from a sensor designed to measure a specific physical quantity (e.g., angular velocity) is used to determine another physical quantity (e.g., the position of the COP) that cannot be directly derived from the original physical quantity (such as angular displacement, angular acceleration, torque, power and angular kinetic energy).

**Figure 8:**
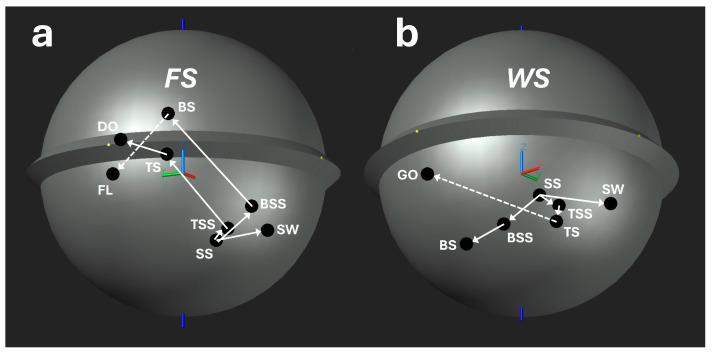
COP transitions between different deliveries (COP positions at the peak torque): (**a**) FS: finger spin; (**b**) WS: wrist spin; SS: side spin; FL: flipper; BS: back spin; BSS: back side spin; SW: swerve; TSS: top side spin; TS: top spin; DO: doosra; GO: googly; solid lines: continuous transition; dashed lines: discontinuous transition.

For the smart cricket ball, the determination of the COP is sufficiently accurate to distinguish between different spin bowling deliveries. For both finger spin (FS) and wrist spin (WS) deliveries (Figure 8a,b), continuous transitions from side spin (SS) to swerve (SW); from side spin (SS) to top side spin (TSS) to top spin (TS) and side spin (SS) to back side spin (BSS) to back spin (BS) can be seen. Even for finger spin (FS), there is a continuous transition from top spin (TS) to doosra (DO), which is not surprising, since the spin axis of the top spin (TS) points +90° to the left (0° = pointing forward), while the doosra (DO) is a hyper-top spin with the spin axis tilted well over 90°. These continuous transitions indicate that the underlying bowling technique remains consistent within both the finger spin (FS) and wrist spin (WS) families. However, within the finger spin (FS) deliveries, there is no direct transition from back spin (BS) to flipper (FL), despite both being back spin deliveries. This is due to the distinct technique employed for the flipper. In executing a flipper, the fingers and thumb spin the ball from a more flexed wrist position, contrasting with the back spin delivery, which relies predominantly on the index and second fingers. Consequently, the flipper technique diverges significantly from the standard finger spin technique, preventing a seamless transition between these two types of deliveries. The same principle applies to wrist spin (WS) deliveries, notably the absence of a continuous transition from top spin (TS) to the googly (GO), despite both involving top spin mechanics. This discontinuity signifies a substantial change in technique. The TS delivery is relatively straightforward, requiring less shoulder flexibility, as the angular velocity vectors of the bowling arm and finger motion are closely aligned. In contrast, the GO delivery imposes greater strain on the shoulder and demands a more complex finger action. The fingers must impart a hyper-top spin with a side spin component, creating a significant angular offset between the finger motion and the bowling arm. This angular discrepancy can be quantified by the dot product of the angular velocity vectors of the bowling arm and the finger motion, illustrating the biomechanical differences between the top spin (TS) and googly (GO) deliveries. This biomechanical complexity explains why there is not a smooth transition between top spin (TS) and googly (GO) and why many spin bowlers can deliver top spin (TS) effectively but relatively few can bowl an effective googly (GO). In fact, both flipper (FL) and googly (GO) have a significantly smaller *efficiency* (52.5% and 57%, respectively) [6] than back spin (BS) and top spin (TS) deliveries (57.5% and 78%, respectively) [6]. The efficiency in this context is a skill performance parameter calculated from the ratio of the actual angular kinetic energy (calculated from the spin torque *T_S_*) to the ideal angular kinetic energy (calculated from the resultant torque *T_R_* if *T_R_* were ideally identical with *T_S_*) [6].

Both discontinuous transitions cross the paths of the opposite continuous transitions (BS to FL crosses the top spin transition, Figure 8a, and TS to GO crosses the back spin transition, Figure 8b). The position of the COP depends on the movement and the bowling technique.

The limitations of this study are related to the data sampling frequency of smart balls. For the smart cricket ball, the data sampling frequency of 815 Hz is sufficient, although the torque data had to be interpolated to achieve a better COP density. In contrast, for the smart football, the data sampling frequency of 1024 Hz was not suitable to calculate the COP. Even interpolation was insufficient. To increase the data sampling frequency from 1024 Hz to an acceptable 7500 Hz, the raw data had to be fitted using sixth-order polynomials.

For the smart football, only a single kick was available to investigate the effectiveness of the COP algorithm for football applications and, if ineffective, to identify ways to improve the data for calculating the COP. Unlike the smart cricket ball test, the smart football test was not intended for validation. To validate the COP algorithm in smart footballs in the future, the data sampling frequency should be at least 7500 Hz, and the ball/sensor coordinate system must be clearly marked on the surface of the ball and aligned with the global coordinate system. The ball should then be kicked in the same way, with comparable translational and angular velocities.

In cricket, the position of the COP is related to how torque is transmitted to the ball. The longer the COP moves along the surface of the ball, the longer time it takes for the transmission of torque. In fast bowling, the COP should travel along the side of the seam where the middle finger is placed rather than along the seam itself [5]. The same principles apply to football, especially for a curved kick. The position of the COP reveals the balance between the Magnus effect needed to curve the ball (optimal COP at the equator) and the appropriate launch angle (optimal COP in the lower hemisphere). This optimal position could even be translated into an auditory biofeedback signal to enhance the learning curve for training curved kicks.

The algorithm presented in this study has the potential to be applied to other smart spherical balls, such as baseballs [7,8], ten-pin bowling balls [9] and basketballs [10,11,12]. This could significantly enhance the understanding and analysis of ball dynamics in various sports. However, the current form of the algorithm is not suitable for oval balls. This is because a normal force applied to the surface of an oval ball typically does not pass through its centre. Examples of instrumented oval balls include smart American footballs [13,14] and smart rugby balls (Gilbert Rugby, Robertsbridge, UK).

## Figures and Tables

**Table 1:** Average spin rates *ω* and torques *T* of the deliveries used for calculating the COP, shown in Figure 6.

Spin Type	Abbr.	Subtype	Code	*ω* (rps)	*T* (Nm)
finger spin	FS	flipper	FL	17.7	0.200
finger spin	FS	backspin	BS	17.4	0.194
finger spin	FS	back-sidespin	BSS	19.5	0.221
finger spin	FS	sidespin	SS	21.6	0.229
finger spin	FS	top-sidespin	TSS	22.6	0.226
finger spin	FS	topspin	TS	16.7	0.137
finger spin	FS	doosra	DO	17.0	0.149
wrist spin	WS	backspin	BS	18.7	0.180
wrist spin	WS	back-sidespin	BSS	24.9	0.230
wrist spin	WS	sidespin	SS	24.1	0.201
wrist spin	WS	top-sidespin	TSS	25.4	0.218
wrist spin	WS	topspin	TS	22.6	0.203
wrist spin	WS	googly	GO	20.0	0.199

## Data Availability

The data presented in this study are available on request from the first author to any qualified researcher who has obtained ethics approval for secondary use of existing data through a consent waiver.
